# The First Bromeligenous Species of *Dendropsophus* (Anura: Hylidae) from Brazil's Atlantic Forest

**DOI:** 10.1371/journal.pone.0142893

**Published:** 2015-12-09

**Authors:** Rodrigo B. Ferreira, Julián Faivovich, Karen H. Beard, José P. Pombal

**Affiliations:** 1 Department of Wildland Resources and Ecology Center, Utah State University, Logan, Utah, United States of America; 2 Laboratório de Ecologia de Populações e Conservação, Universidade Vila Velha, Vila Velha, Espírito Santo, Brazil; 3 División Herpetología, Museo Argentino de Ciencias Naturales “Bernardino Rivadavia”- CONICET, Buenos Aires, Argentina; 4 Departamento de Biodiversidad y Biología Experimental, Facultad de Ciencias Exactas y Naturales, Universidad de Buenos Aires, Buenos Aires, Argentina; 5 Departamento de Vertebrados, Museu Nacional, Universidade Federal do Rio de Janeiro, Rio de Janeiro, RJ, Brazil; Trier University, GERMANY

## Abstract

We describe a new treefrog species of *Dendropsophus* collected on rocky outcrops in the Brazilian Atlantic Forest. Ecologically, the new species can be distinguished from all known congeners by having a larval phase associated with rainwater accumulated in bromeliad phytotelms instead of temporary or lentic water bodies. Phylogenetic analysis based on molecular data confirms that the new species is a member of *Dendropsophus*; our analysis does not assign it to any recognized species group in the genus. Morphologically, based on comparison with the 96 known congeners, the new species is diagnosed by its small size, framed dorsal color pattern, and short webbing between toes IV-V. The advertisement call is composed of a moderate-pitched two-note call (~5 kHz). The territorial call contains more notes and pulses than the advertisement call. Field observations suggest that this new bromeligenous species uses a variety of bromeliad species to breed in, and may be both territorial and exhibit male parental care.

## Introduction

*Dendropsophus* is one of the most taxonomically complex genera of hylids due to its high intraspecific variation and high morphological similarity among most species [1,2,3]. *Dendropsophus* is currently composed of 96 species distributed from Mexico to Argentina [4]. The larval phase of *Dendropsophus* species, when known, is always associated with temporary or lentic water bodies (i.e. ponds and swamps).

Twenty-four of the 96 species of *Dendropsophus* occur along the Atlantic coast of Brazil. The mountainous region of Brazil’s Atlantic Forest is known for its remarkable diversity and endemism of anurans [5,6]. However, the region’s diversity is presumably far from being completely described considering the high rate at which new taxa are discovered [7,8,9].

Rocky outcrops are a unique landscape feature (i.e. mostly granite dome-shaped, shallow soils, rapid water runoff) found across Brazil’s Atlantic Forest. Bromeliads are the dominant plants in these environments [10] and are remarkably important for the local biota. Bromeliads accumulate rainwater between leaves, which provides refuge, moisture, and water for their associated biota [11,12,13].

While surveying for frogs inside bromeliads on rocky outcrops in the municipality of Santa Teresa, we found a distinct treefrog jumping out of an epiphytic bromeliad. Here we describe this new bromeligenous species (i.e. larval phase spent inside bromeliads [14]), including its advertisement and territorial calls, and investigate its phylogenetic relationships to other species in the genus based on molecular data. We compare the new species with the 96 known congeners and comment on its natural history and conservation status.

## Materials and Methods

### Study region

Field research was conducted in and around the Reserva Biológica Augusto Ruschi (REBIO, 19°54’S, 40°32’W, datum = WGS84), Santa Teresa, State of Espírito Santo, Brazil. Sampled sites range from 745 to 922 meters above sea level (m a.s.l.). Sampling sites are in the Atlantic Forest domain, and are classified as montane and sub-montane rainy forest composed of non-deciduous trees [15].

Santa Teresa’s climate is classified as Cwa-Cfa according to Köppen-Geiger’s classification [16]. The dry season is from May to August and the rainy season is from September to April [17]. Mean annual precipitation is 1868 mm with the highest monthly rainfall in November (326 mm) and lowest in June (60 mm) [17]. Mean annual temperature is 20°C, with minimum and maximum monthly temperatures averaging 14.3°C (July) and 26.2°C (January), respectively [18].

### Sampling

We surveyed nine areas with high density of bromeliads during the rainy season of 2012 (August to December) and the dry season of 2013 (June and July). Two of these sites were located onn rocky outcrops and seven sites were located in the forest interior. Four collectors visited each site and actively searched inside bromeliads.

We identified occupied bromeliad to species level and determined their location (epiphyte or ground). We also measured plant diameter, plant height, the number of leaves, and height off the ground. We used Pearson’s chi-square exact test (χ^2^) to compare characteristics of bromeliads occupied and unoccupied by the new species using a Monte Carlo simulation based on 999 replicates using the package *MASS* [19].

We euthanized the frogs by ventral application of 7.5% to 10% benzocaine, fixed them in 10% formalin, and preserved them in 70% ethanol within one to five days of fixation [20,21]. Collected specimens were deposited in the collections of Museu Nacional, Universidade Federal do Rio de Janeiro (MNRJ), State of Rio de Janeiro and the Museu de Biologia Mello Leitão (MBML), State of Espírito Santo, both in Brazil. Prior to fixation, some specimens had tissue samples extracted and stored in 92% ethanol for DNA extraction. Some tadpoles collected from the bromeliads were raised in captivity until metamorphosis to confirm species identification. Vouchers of bromeliads were deposited in the herbarium of MBML.

This study was carried out in strict accordance with the recommendations of the Guidelines for euthanasia of animals from the veterinary medical association of both Brazil and the United States of America. Research protocols were approved by the Instituto Chico Mendes de Conservação da Biodiversidade (ICMBio, Permit Number: 28607–3) and the Institutional Animal Care and Use Committee of Utah State University (IACUC-USU, Permit Number 2002).

### Morphology

Based mostly on molecular data, Faivovich et al. [22] recognized nine species groups of *Dendropsophus*: the *D*. *columbianus*, *D*. *garagoensis*, *D*. *labialis*, *D*. *leucophyllatus*, *D*. *marmoratus*, *D*. *microcephalus*, *D*. *minimus*, *D*. *minutus*, and *D*. *parviceps* groups. Herein, we compared 12 individuals of the new species with all the known species in the genus. Specimens of 44 species used for direct comparisons as well as their museum numbers are listed in S1 File.

We used an ocular micrometer in a Zeiss stereomicroscope for most measurements and a caliper with 0.1 mm precision for measurement of snout-vent length (SVL). We followed Duellman [23] for morphological terminology: SVL, HL (head length), HW (head width), ED (eye diameter), TD (tympanum diameter), IOD (interorbital distance), END (eye-nostril distance), IND (internarial distance), THL (thigh length), TL (tibia length), and FL (foot length). Descriptions of the coloration of living specimens are based on digital color photographs taken in the field.

### Call recording

We recorded calls using a Marantz PMD-660 digital recorder attached to a Sennheiser ME 64/K6p external directional microphone. We digitalized the calls at a resolution of 16 bits and a sampling rate of 48 kHz. For the bioacoustics analyses, we used Audacity 2.0.5 [24] and the package *Seewave* [25].

We evaluated the following parameters: number of pulses per note; number of notes per call; call, note and pulse duration (milliseconds = ms); interval between calls (ms); and dominant frequency of the note (Hertz = Hz). Advertisement call terminology follows Duellman and Trueb [26]. Call categorization follows Toledo et al. [27]. We deposited the call recordings in the Coleção Científica de Vocalizações de Anfíbios Anuros do Museu Nacional–Universidade Federal do Rio de Janeiro (MNVOC 048/01-06).

We performed one-way analysis of variance (ANOVA) to compare interval of males calling in chorus and alone, and to compare duration and frequency between notes of the advertisement call. Mean ($\bar{X}$) ± standard deviation (SD) are presented. We performed all the above statistical analyses in the version 3.0.3 of R [28].

### Molecular analysis

We extracted DNA from the holotype (MNRJ 85852) and two paratopotypes (MNRJ 85854 and MNRJ 85857) of the new species (GenBank accession number: KT962842, KT962843, and KT962844). We sequenced the complete 12S rRNA gene and a fragment of the 16S rRNA gene, including the intervening valine-tRNA, using the same primers employed by Faivovich et al. [22]. DNA extraction, amplification, and sequencing methods are those described in a recent paper by Faivovich et al. [29]. We sequenced all samples in both directions. Chromatograms obtained from the automated sequencer were read and contigs made using the sequence editing software SEQUENCHER 3.0 (Gene Codes, Ann Arbor, MI, USA). Complete sequences were edited with BioEdit [30].

We performed a preliminary phylogenetic analysis including a broad diversity of hylids, which indicated that the new species belonged to the genus *Dendropsophus*. We used sequences of the mitochondrial genes 12S+trna^VAL^+16S and the dataset of Rivera-Correa & Orrico [31] to explore the relationship of the new species to other species in the genus. This dataset included sequences of 37 of the 96 known species of *Dendropsophus* [4], including exemplar species of all species groups currently recognized. Furthermore, it includes 11 outgroup taxa of the genera *Lysapsus*, *Phyllodytes*, *Pseudis*, *Scarthyla*, *Scinax*, *Sphaenorhynchus*, and *Xenohyla*.

We generated static alignments in MAFFT [32] with Q-INS-i strategy (secondary structure of RNA is considered). We performed maximum parsimony analyses using T.N.T Willi Henning Society Edition [33]. We did the searches using the new technology under level 50, which included sectorial, tree drift and tree fusing [34], and the driven search to hit the best length 100 times. We estimated Parsimony Jackknife absolute frequencies [35] using new technology as well as requesting 5 hits with driven searches, for a total of 1000 replicates.

We performed Bayesian analyses using MrBayes 3.2 [36] as implemented in the CIPRES web based platform [37]. The models of molecular evolution were determined for the combined data by gene using Partition Finder 1.01 [38]. We used the GTR+I+G model for 12S and 16S. Bayesian analyses included four independent runs with three heated chains and one cold chain in each run. The MCMC chains were run for 80,000,000 generations and sampled every 1,000 generations. We examined trace plots and effective sample size (ESS) in Tracer v1.5 to determine MCMC mixing and convergence. We removed trees from the first 25% of the samples as burn-in. A consensus of the post-burning trees was visualized in FigTree v1.3.1.

### Nomenclatural acts

The electronic edition of this article conforms to the requirements of the amended International Code of Zoological Nomenclature (ICZN), and hence the new name contained herein is available under that Code from the electronic edition of this article. This published work and the nomenclatural acts it contains have been registered in ZooBank, the online registration system for the ICZN. The ZooBank LSID (Life Science Identifier) can be resolved and the associated information viewed through any standard web browser by appending the LSID to the prefix ‘‘http://zoobank.org/”. The LSID for this publication is: urn:lsid:zoobank.org:pub:655D9BD9-A700-47C6-8C70-418A2929C511. The electronic edition of this work was published in a journal with an ISSN, and has been archived and is available from the following digital repositories: PubMed Central, LOCKSS.

## Results

**Fig 1:**
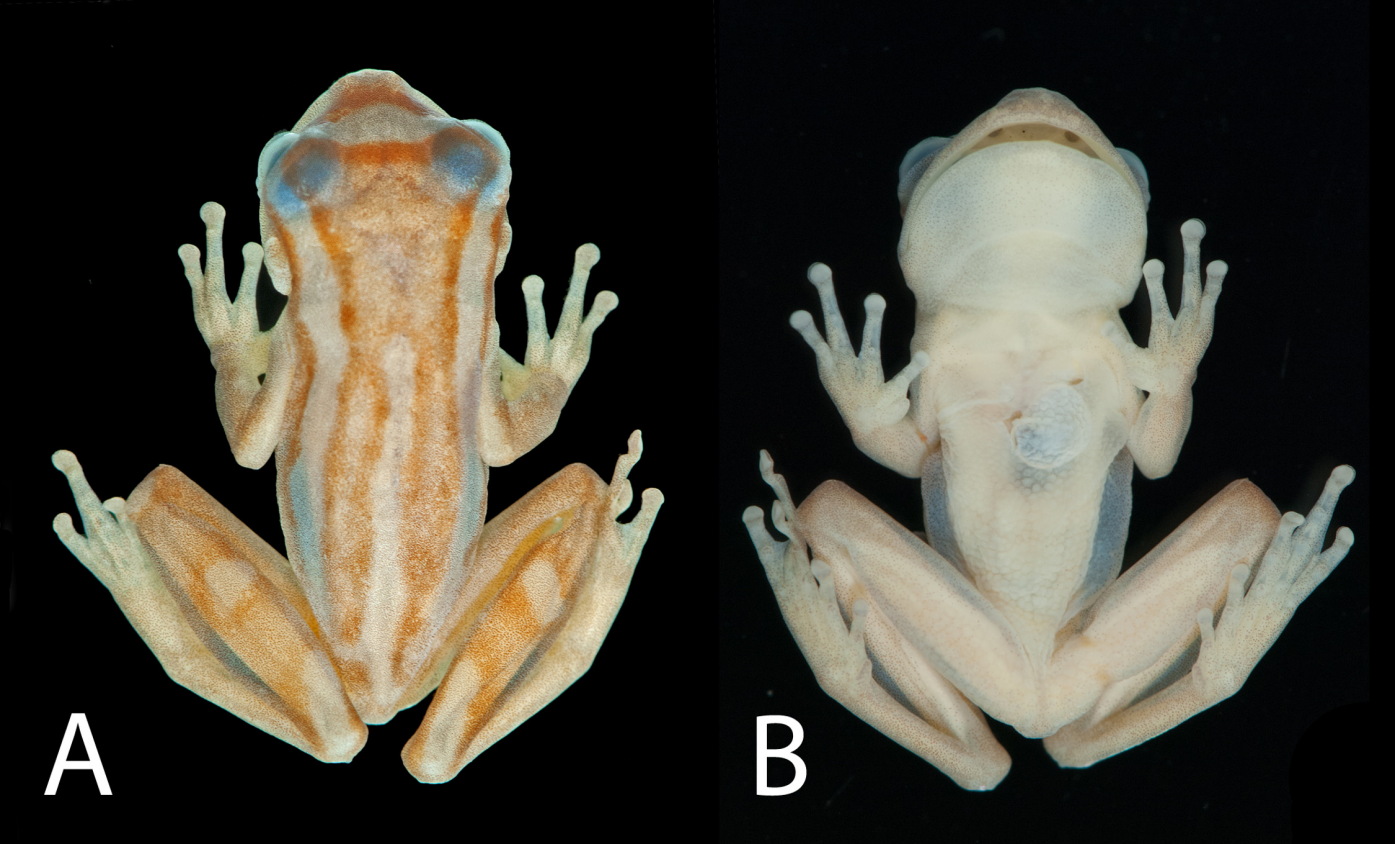
Holotype of *Dendropsophus bromeliaceus* sp. nov.. (A) Dorsal and (B) ventral views (MNRJ 85852, SVL 16.7 mm).

**Fig 2:**
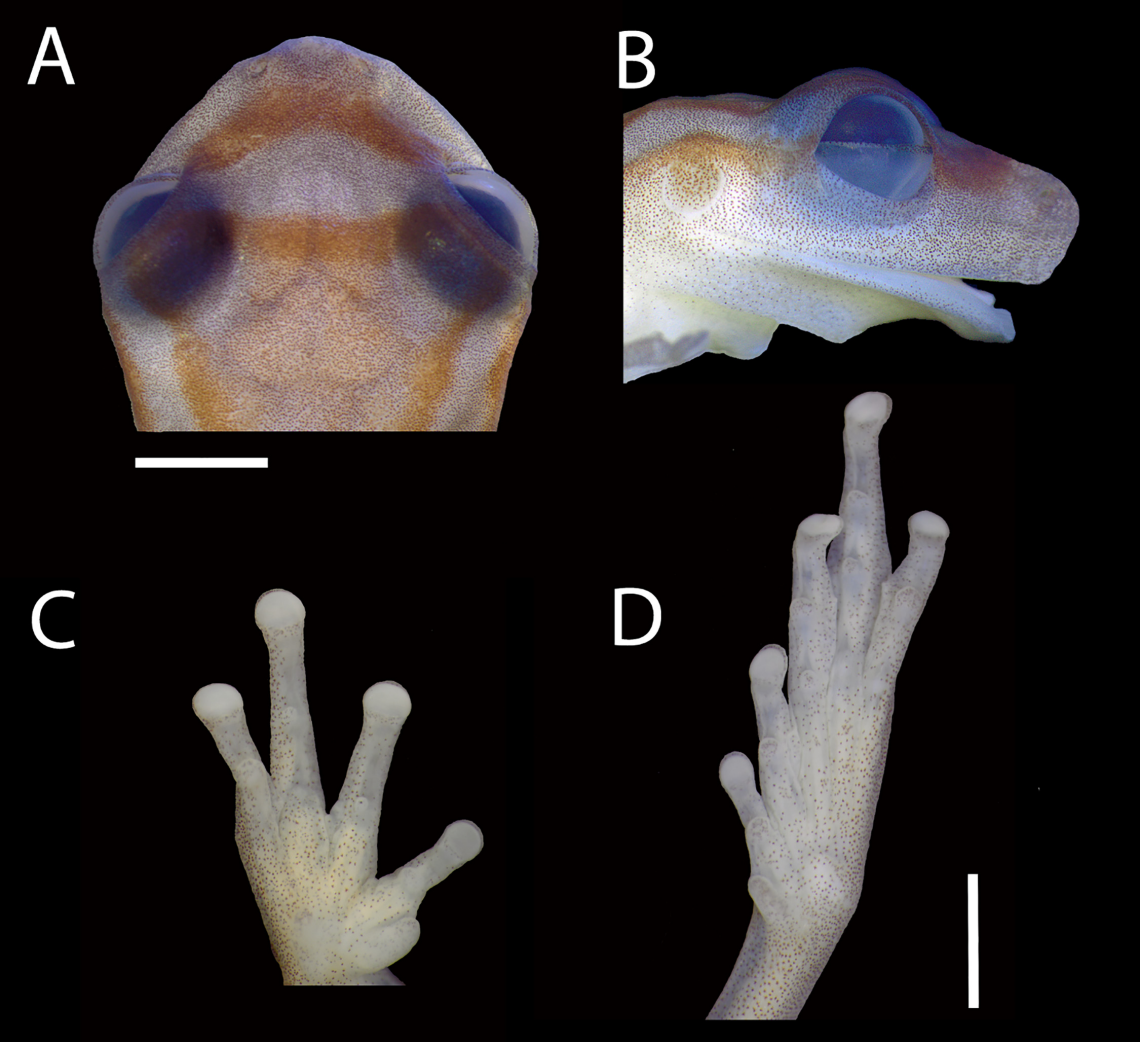
Holotype of *Dendropsophus bromeliaceus* sp. nov.. (A) Dorsal and (B) lateral views of head, (C) palmar view of left hand, and (D) plantar view of right foot (MNRJ 85852). Scale bar = 2 mm.

**Fig 3:**
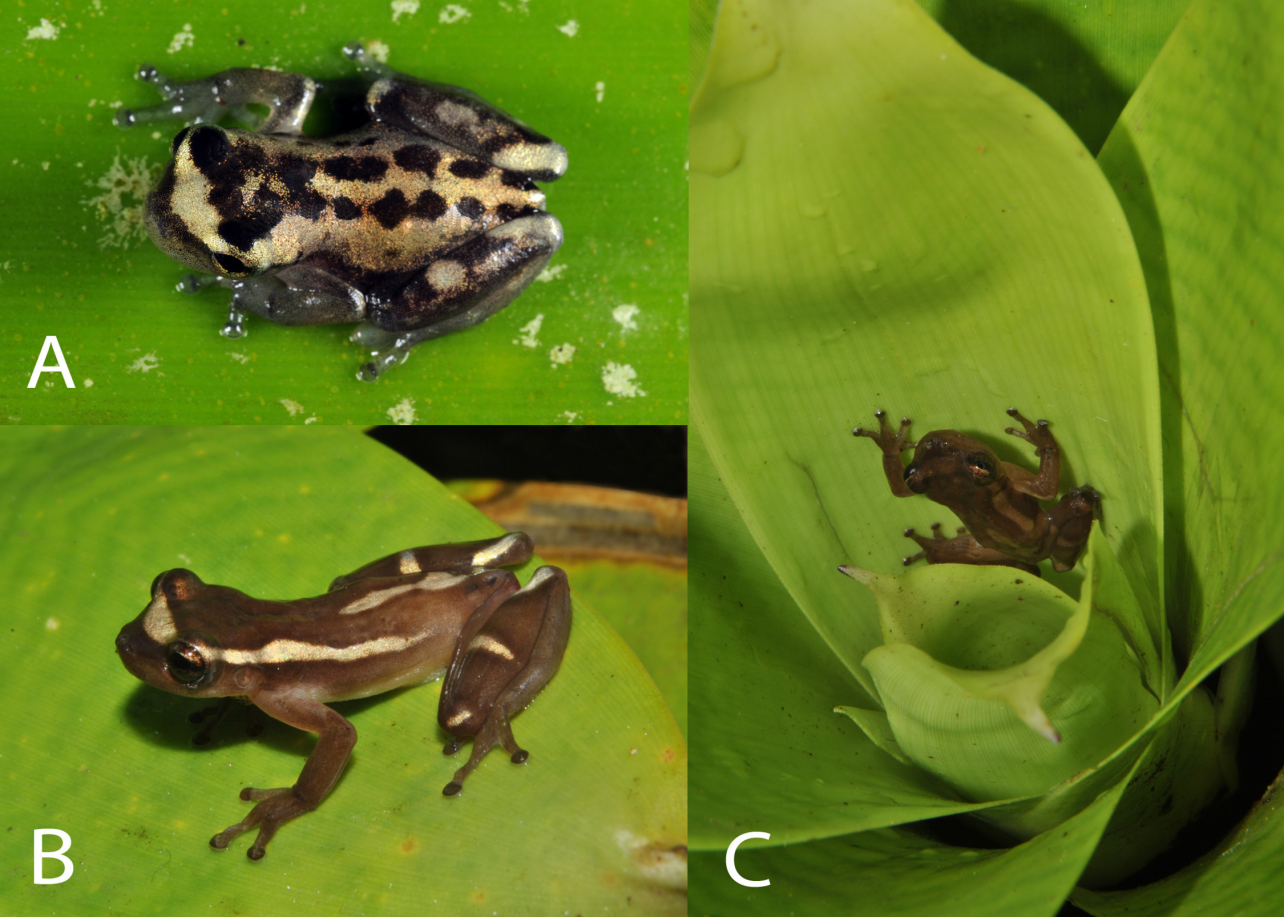
*Dendropsophus bromeliaceus* sp. nov. in life. (A) froglet (MNRJ 85855), and (B and C) male paratopotype (MBML 7712).

*Dendropsophus bromeliaceus* sp. nov. (Figs 1, 2 and 3) urn:lsid:zoobank.org:pub:6C332786-14DC-4314-B064-47B54B81A977

**Etymology.** The specific epithet “bromeliaceus” refers to the reproductive habit of the new species, which deposits eggs in bromeliads and spends the larval phase in the rainwater accumulated in these plants. The suffix “aceus” is Latin, meaning “belonging to”.

**Common names.** We suggest Teresensis’ bromeliad treefrog or Pererequinha-de-bromélia-teresensis (in Portuguese). Teresensis refers to the people born in the municipality of Santa Teresa.

**Holotype.** MNRJ 85852, adult male, collected in the surroundings of the Reserva Biológica Augusto Ruschi (19°54’27”S, 40°31’05”W; 878 m a.s.l.), Santa Teresa, State of Espírito Santo, Brazil, on 3 December 2012 by R. B. Ferreira and team (see Acknowledgments).

**Paratopotypes.** MNRJ 85848, 85851, 85854, 85856, males, collected on 3 December 2012; MNRJ 85857, 85859, males, collected on 10 December 2012; MNRJ 85860–61, male and female respectively, collected on 15 December 2012; MNRJ 85862, male, collected on 10 January 2013; MBML 7712, male, collected on 1 July 2013. Collected in the vicinity of Associação do Banestes (19°55’52”S, 40°35’18”W; 764 m a.s.l.): MNRJ 85863, male, collected on 2 October 2012. These specimens collected by R. B. Ferreira and team. Collected in Sítio Vista Linda (19°48’23”S, 40°33’13”W; 905 m a.s.l.): MBML 7722, 7724, males; MBML 7720–21, 7726, females; MBML 7727, unsexed, collected on 14 April 2015 by Fernanda C. F. Lirio.

**Diagnosis.** *Dendropsophus bromeliaceus* sp. nov. is diagnosable from all known congeners by the following combination of characters: (1) small size (male SVL 16.8 ± 6.6 mm (16.1–18.4 mm)); (2) framed dorsal color pattern; (3) visible tympanum; (4) short webbing between toes IV-V; (5) light cream belly; (6) reduced axillary membrane; (7) finger and toe discs almost rounded; (8) absence of cloacal sheath covering entirely the cloacal opening; (9) absence of a white spot under the eye; (10) absence of two whitish lines from snout to sacral region; and (11) advertisement call composed of two moderate pitched notes (~5 kHz) with the first note containing 3–6 pulses, and the second note containing 4–8 pulses. Ecologically, *Dendropsophus bromeliaceus* sp. nov. can be distinguished from all other known congeners by having a tadpole phase associated with rainwater that accumulates in bromeliads.

**Description of the holotype.** Adult male, SVL 16.7 mm; body moderately robust; head wider than body, widest below eyes, wider than long (HW/HL 1.1); head length representing 35.9% of SVL; snout short, nearly truncate in dorsal and lateral views; eye-nostril distance smaller than eye diameter (END/ED 0.89); *canthus rostralis* indistinct, almost straight; loreal region slightly concave; internarial area slightly depressed; nostrils not protuberant, directed dorsolaterally; interorbital area flat; IOD/ED 1.31; IOD/HW 0.37; eyes large and protuberant (ED/HL 0.31; ED/HW 0.28); pupil horizontal, elliptical; nictitating membrane transparent, its free margin pigmented in the same pattern of the eyelid; distinct supratympanic fold, semi-circular from eye to above the arm insertion; tympanum small (TD/HL 0.16), distinct, rounded, separated from the eye; choanae medium size, oval; vomerine odontophores very small; tongue large, cordiform, slightly notched behind; vocal slits long, extending from midlateral base of tongue to almost the angle of jaws; vocal sac, medium sized, and subgular. Arm slender; forearm more robust than upper arm; arm without fold or fringe; axillary membrane reduced; fingers slender; relative length of fingers II<III≈V<IV; discs nearly round, small; disc of finger II smaller than others; subarticular tubercles round, most prominent on fingers II and IV; supernumerary tubercles present; a large elliptical inner metacarpal tubercle; a bifid, medium sized outer metacarpal tubercle; short digital webbing; webbing formula II trace III 2^-—^3^-^IV3^+^—3^+^V; nuptial pad unpigmented, glandular acini on the proximal surface of finger II. Hind limbs long and slender (THL/SVL 0.62); no tarsal tubercle or fimbria; toes moderately slender; relative length of toes I<II<III≈V<IV; inner metatarsal tubercle small, approximately oval; outer indistinct; toe discs small, round, similar size to the finger discs; subarticular tubercles protruding; tubercles on toes III and IV round; short digital webbing; webbing formula I trace II 3^-^—3^1/2^ III 3^+^—3^1/2^IV 3 ^½^ —3^-^V. Dorsal skin surfaces, flanks, and throat smooth; skin on belly and ventral surfaces of thighs granular; cloacal opening directed posteriorly, covered by a small sheath dorsally, and surrounded by cloacal tubercles, including a pair of tubercles below the cloacal opening.

**Color of the holotype in preservative.** General dorsal color light brown; cream, wide dorsolateral stripe from behind the eye to inguinal region; a cream mid-dorsal stripe from level of arm insertion to cloacal opening; cream blotch similar to a triangle shape, with apex directed towards nostrils from slightly behind nostrils to the anterior part of eyelids; lateral areas of the head and flank cream; thigh cream; tibia light brown with two cream blotches; forelimbs grayish. Undersurfaces light cream; gular region, palm of hand, undersurfaces of the thigh and foot with blackish little spots, spots more dispersed in gular region. Iris black.

**Variation.** Measurements of the type series are shown in Table 1. The only female (MNRJ 85861) was larger than the males (Table 1). Morphology and color pattern are generally concordant with the holotype. The dorsal color pattern in life can be lighter or darker; two specimens are very light, with the dorsal line pattern almost indistinct. The dorsolateral and mid-dorsal stripes may be longer or shorter than in the holotype; the dorsolateral stripe can start from the eye (n = 6), and the mid-dorsal stripe can be short, interrupted or reduced to a blotch; the blotch between and anterior to the eyes on the snout can be almost triangular and wider in its medial portion. The froglets (MNRJ 85849, 85850, 85853, 85855, and 85858) have a mean SVL of 10.7 mm (range: 9.6–11.4 mm).

**Table 1:** Measurements of the type series of *Dendropsophus bromeliaceus* sp. nov.. Values in millimeters presented as mean ± standard deviation (range).

	Holotype	Paratopotypes	
Measurement	Male	Males (n = 10)	Female (n = 1)
Snout-vent length (SVL)	16.7	16.8 ± 6.6 (16.1–18.4)	20.1
Head length (HL)	6.0	5.9 ± 3.52 (5.4–6.5)	6.5
Head width (HW)	6.6	6.4 ± 2.27 (6.0–6.9)	7.2
Eye diameter (ED)	1.9	1.8 ± 1.31 (1.7–2.1)	2.0
Tympanum diameter (TD)	1.0	0.8 ± 1.41 (0.6–1.0)	1.5
Interorbital distance (IOD)	2.5	2.2 ± 1.37 (2.1–2.5)	2.7
Eye-nostril distance (END)	1.7	1.4 ± 1.41 (1.3–1.7)	1.7
Internarial distance (IND)	1.3	1.2 ± 0.09 (1.1–1.4)	1.4
Thigh length (THL)	8.4	8.3 ± 3.25 (7.8–8.6)	10.5
Tibia length (TL)	10.5	8.8 ± 4.59 (7.9–9.3)	11.0
Foot length (FL)	6.4	7.0 ± 4.73 (6.1–7.5)	8.4

**Coloration in life** (based on paratopotypes MBML 7712, MNRJ 85857, 85859; Fig 3). General dorsal color dark brown; golden dorsolateral stripe from behind the eye to inguinal region; a golden mid-dorsal bar from level of arm insertion to cloacal opening; triangle-shaped golden blotch, with apex directed towards nostrils from slightly behind nostrils to the anterior part of eyelids; lateral areas of head and flank brown; thigh brown; tibia brown with two or three golden blotches; forelimbs brown; hidden surfaces of thighs brown or light orange. Undersurfaces light cream; gular region, palm of hand, undersurfaces of thigh and foot cream punctuated with black, more dispersed in gular region. Iris copper.

Juvenile froglets have a distinctly different coloration in life compared to adults (Fig 3). Dorsal surfaces of head and body golden; two dorsolateral bars formed by interconnected black blotches. Lateral view of head and flank black; thigh black; tibia black with two golden blotches; forelimbs dark grey.

**Comparison with other species.** The framed dorsal color pattern distinguishes *Dendropsophus bromeliaceus* from species of the *D*. *microcephalus* group, except the *D*. *decipiens* clade. Because of the framed dorsal color and small size, the *D*. *decipiens* clade (except *D*. *berthalutzae)* is superficially similar to *D*. *bromeliaceus*. The new species differs from *D*. *decipiens*, *D*. *haddadi*, and *D*. *oliveirai* by short webbing between toes IV-V (in *D*. *decipiens*, *D*. *haddadi*, and *D*. *oliveirai* webbing reaches the disc on the fifth toe). The new species differs from *D*. *berthalutzae* by the absence of an X-shaped mark on the dorsum, a medium sized vocal sac, and short webbing between toes IV-V (*D*. *berthalutzae* has an X-shaped mark on the dorsum [39,40]). The new species is distinguished from species of the *D*. *rubicundulus* clade (*sensu* [22]) by its framed dorsal color (dorsum dark green in life and violet in preservative in the *D*. *rubicundulus* clade [22,41]). Additionally, the new species can be distinguished from *D*. *araguaya*, *D*. *cachimbo*, *D*. *cerradensis*, and *D*. *elianeae* by its smaller size (male SVL combined 18.9–25 mm [41,42,43]). *Dendropsophus bromeliaceus* is smaller than *D*. *bipunctatus*, *D*. *coffeus*, *D*. *gryllatus*, *D*. *juliani*, *D*. *leali*, *D*. *minusculus*, *D*. *phlebodes*, *D*. *rhodopeplus*, *D*. *robertmertensi*, and *D*. *riveroi* (combined male SVL 19.0–25.5 mm [40,44,45,46,47,48]). The new species differs from *D*. *branneri* and *D*. *werneri* (*sensu* [22]), by the absence of a white spot below the eye [2,40]. *Dendropsophus meridianus*, *D*. *nanus*, *D*. *sanborni*, and *D*. *walfordi* have a dorsal pattern with longitudinal stripes or points [40,49]. *Dendropsophus bromeliaceus* differs from *D*. *pseudomeridianus*,by the absence of two whitish lines from snout to sacral region [50].*Dendropsophus bromeliaceus* can be distinguished from *D*. *cruzi* by its medium sized vocal sac and short webbing between toes IV-V (*D*. *cruzi* has a large vocal sac and webbing on the fifth toe to the disc [2]). The new species differs from *D*. *joannae* by lacking bright yellow coloration on the dorsal surfaces of finger and toe discs [51]. The new species can be distinguished from *D*. *mathiassoni* by its dorsal pattern (absence of a distinct dorsal pattern in *D*. *mathiassoni* [52]). The new species is distinguished from *D*. *ozzyi*, *D*. *reichlei*, and *D*. *shiwiarum* by its reduced axillary membrane (developed in these species [46,53,54]). In addition, the new species can be distinguished from *D*. *ozzyi* and *D*. *shiwiarum* by its finger and toe discs near rounded (pointed discs in *D*. *ozzyi* and *D*. *shiwiarum* [53,54]).

*Dendropsophus bromeliaceus* differs by its smaller size from species of the *D*. *columbianus* group (combined SVL 24.6–35.8 mm of *D*. *bogerti*, *D*. *carnifex*, *D*.*columbianus*, and *D*. *norandinus* [52,55,56]), the *D*. *garagoensis* group (combined SVL of males 21.3–31.5 mm of *D*. *garagoensis*, *D*. *padreluna*, *D*. *praestans*, and *D*. *virolinensis* [57,58,59]), the *D*. *labialis* group (combined SVL 26.4–42.0 mm of *D*. *labialis*, *D*. *luddeckei*, and *D*. *meridensis* [52,60,61]), the *D*. *marmoratus* group (combined SVL of males 30.0–45.0 mm in *D*. *acreanus*, *D*. *dutrai*, *D*. *marmoratus*, *D*. *melanargyreus*, *D*. *novaisi*, *D*. *seniculus*, and *D*. *soaresi* [40,62,63,64,65,66]), and the *D*. *leucophyllatus* group (combined males SVL 20.0–40.0 mm [40,56,65,67,68]). Additionally, the new species is distinguished from species of the *D*. *columbianus* group by its light cream belly (bellies are flecked, marbled, or yellow in the *D*. *columbianus* group [55,56]); from the *D*. *labialis* and *D*. *marmoratus* groups by its framed dorsal color (green in life and in preserved specimens of the *D*. *labialis* group, and lichenous, both in life and in preserved specimens of the *D*. *marmoratus* species group [53]). Additionally, *D*. *bromeliaceus* is distinguished from the *D*. *leucophyllatus* group by the absence of a pair of oval pectoral glands (except in *D*. *anceps*) and no vivid colors on the hidden surfaces of thighs, groin, and webbing (presence of pectoral glands and vivid flash color in the other species [31]).

*Dendropsophus bromeliaceus* differs from the *D*. *parviceps* group by its framed dorsal color pattern (the *D*. *parviceps* group is lichenous in life and preserved specimens; see [53]). Additionally, *D*. *bromeliaceus* is distinguished from *D*. *bokermanni*, *D*. *brevifrons*, *D*. *luteoocellatus*, *D*. *microps*, *D*. *piauiniensis*, *D*. *subocularis*, and *D*. *timbeba* by the absence of blotches, spots, or bars on the thighs and groin; and from *D*. *bokermanni*, *D*. *brevifrons*, *D*. *gaucheri*, *D*. *koechlini*, *D*. *luteoocellatus*, *D*. *microps*, *D*. *parviceps*, and *D*. *subocularis* by the absence of a suborbital bar [69,70,71]. It further differs from *D*. *frosti*, *D*. *grandisonae*, *D*. *luteoocellatus*, *D*. *microps*, *D*. *pauiniensis*, *D*. *ruschi*, and *D*. *subocularis* by its smaller size (combined SVL 20.8–33.0 mm [40,61,69,71,72,73,74]). It differs from *D*. *schubarti* by its smaller eye (HL/ED 3.5 versus 2.5 in *D*. *schubarti*) and larger tympanum (HL/TD 4 versus 9.5 in *D*. *schubarti*).

The new species is distinct from species of the *D*. *minutus* group by the absence of a cloacal sheath covering the cloacal opening entirely (present in the *D*. *minutus* group [53]) and by the absence of white supracloacal and tarsal lines (present in species of the *D*. *minutus* group [53]). These same traits also distinguish the new species from *D*. *stingi* and *D*. *aperomeus* [1], which were recently considered to belong to the *D*. *minutus* group [75]. These traits also distinguish *D*. *bromeliaceus* from *D*. *amicorum*, which has white supracloacal and tarsal lines similar to species of the *D*. *minutus* group, although currently not assigned to any species group [4,76,77]. *Dendropsophus bromeliaceus* further differs from *D*. *amicorum*, *D*. *limai*, and *D*. *stingi* by its smaller size (combined SVL 19–26.2 mm [39,76,77,78]).

The new species is distinguished from *Dendropsophus minimus* by its clearly visible tympanum and absence of rostral white line (in *D*. *minimus* the tympanum is concealed and has a rostral white line [79]). *Dendropsophus bromeliaceus* differs from *D*. *miyatai* by its clearly visible tympanum and absence of bright red and yellow dorsal color pattern in life (in *D*. *miyatai* the tympanum is concealed and the dorsal color pattern is bright red and yellow [80]).

Currently, *Dendropsophus amicorum*, *D*. *battersbyi*, *D*. *haraldschultzi*, *D*. *stingi*, *D*. *tintinnabulum*, and *D*. *yaracuyanus* are not included in any species group (see [4,22]). *Dendropsophus amicorum* and *D*. *stingi* were compared to the new species earlier in the text. The new species is easily distinguished from *D*. *battersbyi*, *D*. *tintinnabulum*, and *D*. *yaracuyanus* by its smaller size (combined SVL 19.0–36.6 mm [61,77,81]). *Dendropsophus haraldschultzi* has tuberculate skin on dorsal surfaces, more dense on the hand, and dark longitudinal stripes extending from the interorbital region to the groin [39].

**Fig 4:**
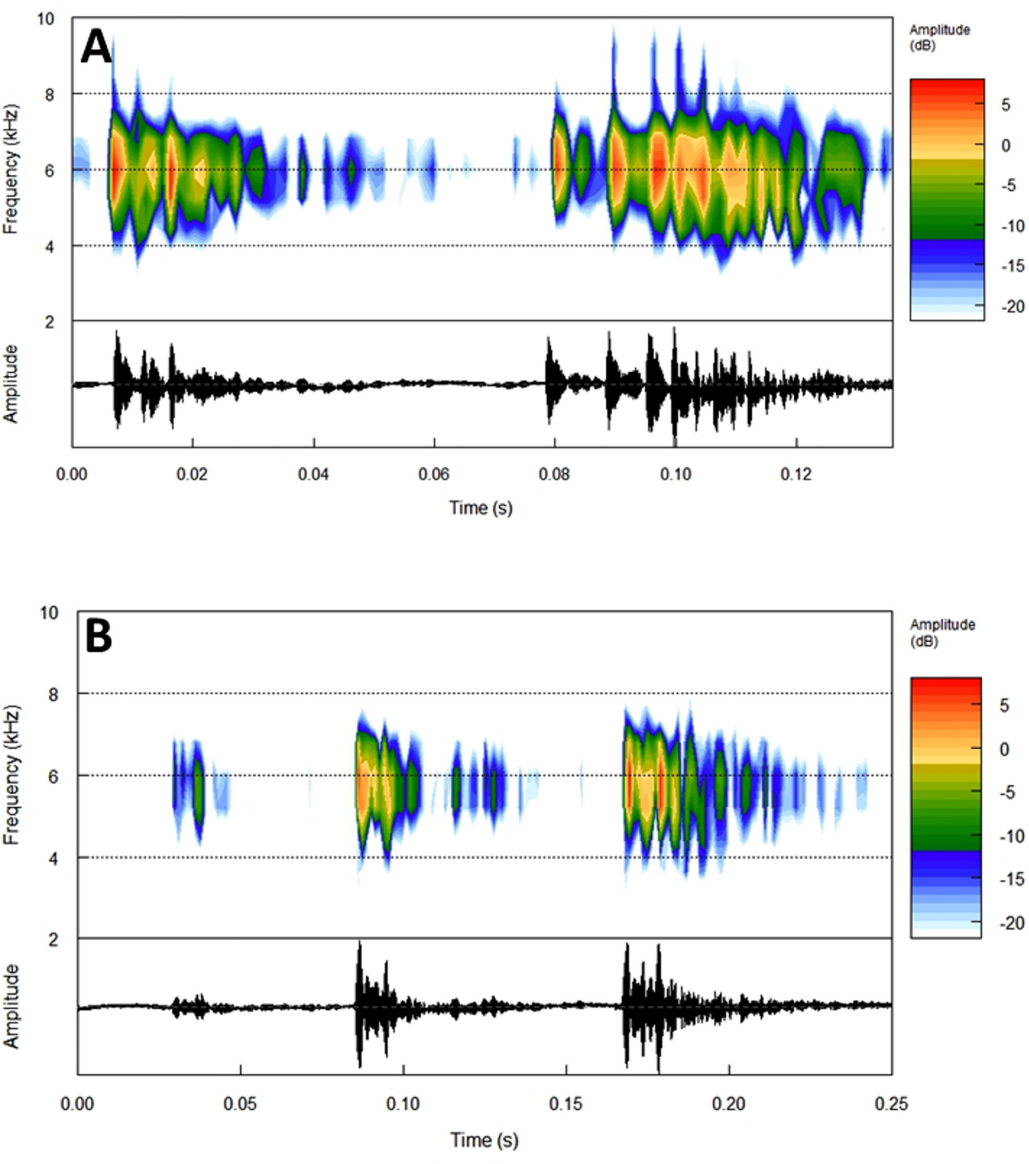
Calls of *Dendropsophus bromeliaceus* sp. nov.. (A) Advertisement call and (B) territorial call with spectrogram (above) and oscillogram (below) (MNVOC 048/01-06). Air temperature was around 23.2°C.

**Vocalization.** The advertisement and territorial calls were recorded at the type locality on 12 and 15 December 2012. Mean air temperature was 23.2 ± 1.2°C and mean relative humidity was 98 ± 3.2% during both recordings. Analyses of the advertisement call are based on 28 calls of four males. The advertisement call consists of a two-note call, and has a mean duration of 1112 ± 87 ms (range: 958–1294 ms). The first note (Note I) contains 3–6 pulses ($\bar{X}$ = 4.27 ± 0.827) and has a mean duration of 225 ± 67 ms (range: 119–362 ms). The second note (Note II) contains 4–8 pulses ($\bar{X}$ = 5.58 ± 0.945) and has a mean duration of 261 ± 51 ms (range: 182–379 ms). Note I is always shorter and with fewer pulses than Note II (F_1,62_ = 5.9; *P* < 0.018). The highest amplitude peak is the first or second pulse in Note I, while it lies in the third or the fourth pulse in Note II (n = 28 calls; 4 males). The spectrogram shows no harmonic structure (Fig 4A), implying that dominant and fundamental frequencies are similar. The dominant frequency is similar between the two notes (F_1,66_ = 1.2; *P* < 0.276) and ranges between 4.8–5.6 kHz ($\bar{X}$ = 5.2 ± 0.2).

The intervals between calls were different in males calling singly and in choruses (F_1,36_ = 23.5; *P* < 0.001). In a chorus, the mean interval was 9.8 ± 2.3 s (range: 7.3–14.0 s; n = 19 calls). Single males had mean intervals of 6.8 ± 1.5 s (range: 4.3–9.5 s; n = 19 calls). Therefore, males in choruses had fewer calls per minute (range = 6–7 calls) compared to males calling alone (range = 8–9 calls). Males in chorus seem to avoid overlapping calls.

Between advertisement calls, *Dendropsophus bromeliaceus* sp. nov. emitted a more complex call consisting of three to four notes (Fig 4B). This call was only emitted by males in neighboring bromeliads, suggesting it was a territorial call. A total of six of these calls (emitted by two males) were analyzed. Territorial calls have a mean duration of 1871 ± 248 ms (range: 1630–2270 ms). Because the first and second notes of the four-note calls have a similar structure, the data presented below concern three-note calls. The first note (Note I) contains 1–3 pulses and has a mean duration of 167 ± 75 ms (range: 100–300 ms). The second note (Note II) has 3–4 pulses and a mean duration of 205 ± 36 ms (range: 150–250 ms). The third note (Note III) has 4–6 pulses and a mean duration of 313 ± 87 ms (range: 210–440 ms). The mean dominant frequency is 5.4 ± 73 kHz (range: 5.3–5.4 kHz).

**Fig 5:**
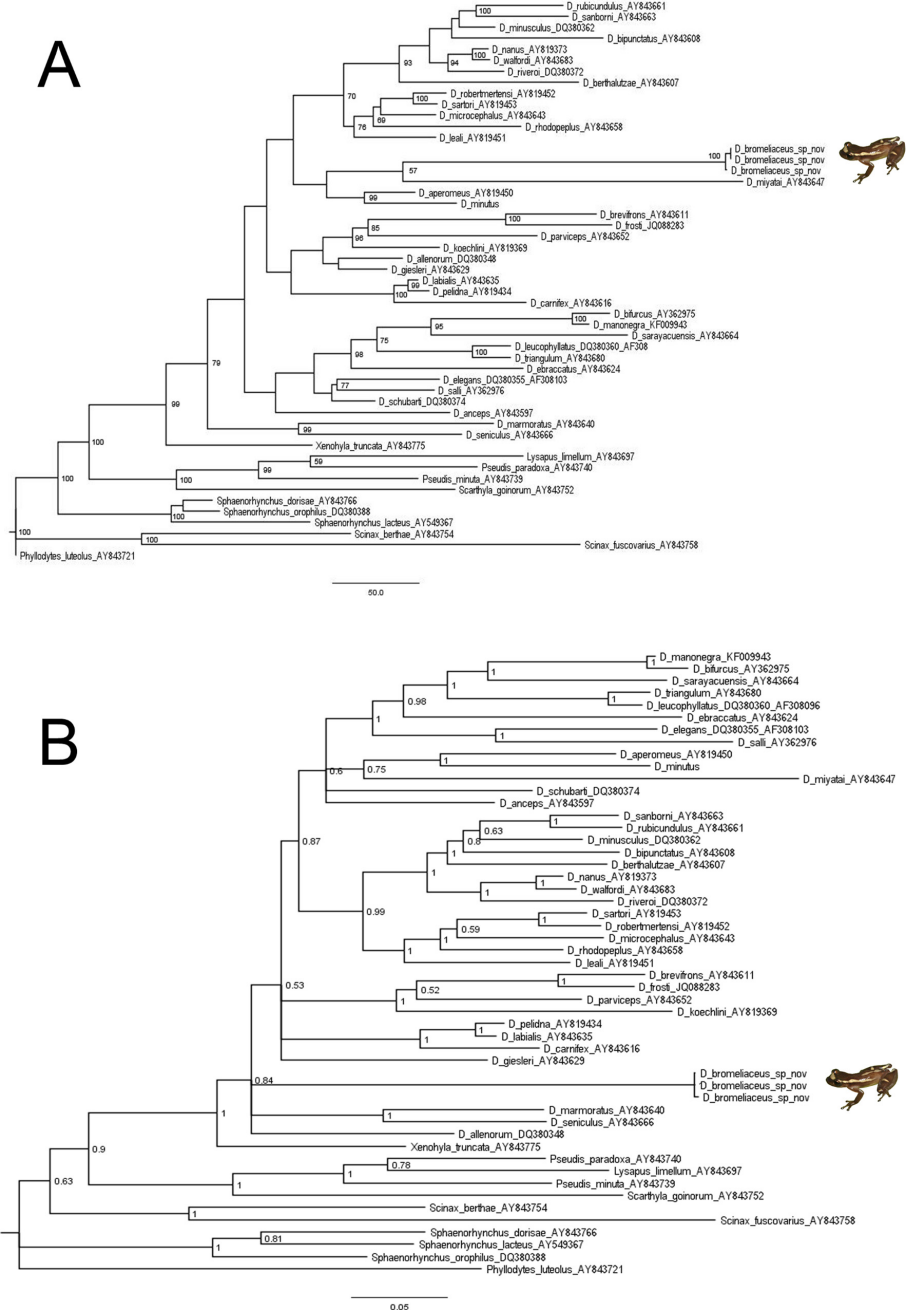
Phylogenetic relationship of *Dendropsophus bromeliaceus* sp. nov.. (A) Maximum parsimony tree; numbers below nodes indicate Jackknife values > 50% and (B) Bayesian tree; numbers below nodes indicate posterior probability. Both results inferred from the mitochondrial genes 12S+trna^VAL^+16S (see methods).

**Phylogenetic relationships.** *Dendropsophus* is monophyletic in both parsimony and Bayesian analyses (Jackknife = 79%; posterior probability = 0.84). *Dendropsophus* is composed of several well-supported (Jackknife > 70%; posterior probability > 0.95) subclades, but the relationships among them need further investigation. The phylogenetic placement of *D*. *bromeliaceus* sp. nov. is still unclear; in the most parsimonious trees *D*. *bromeliaceus* sp. nov. is the sister taxon of *D*. *miyatai* with 57% Jackknife support (Fig 5A). In the Bayesian tree, the new species is grouped in a polytomy at the basal node of *Dendropsophus* (Fig 5B).

**Fig 6:**
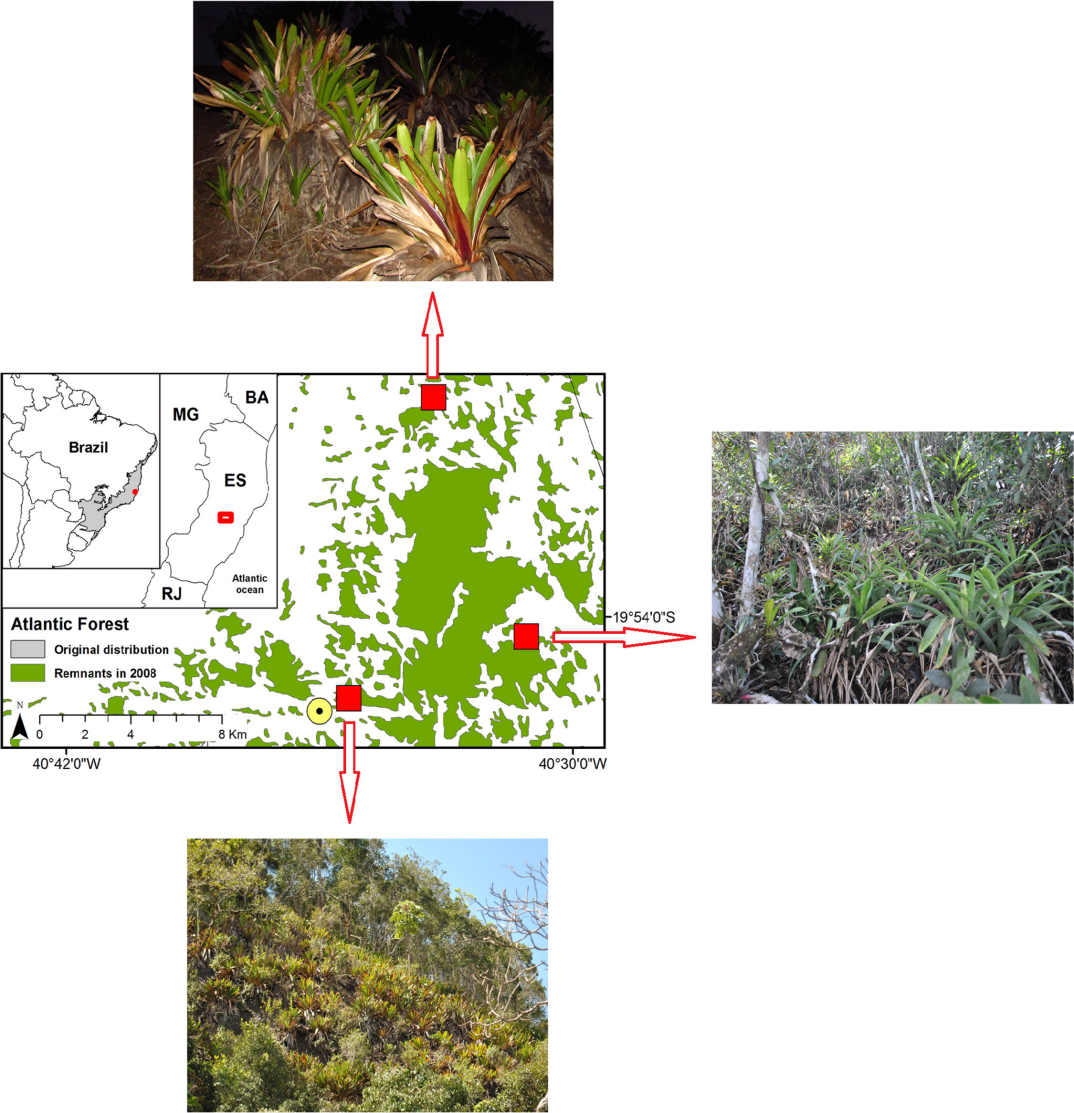
Geographic distribution of *Dendropsophus bromeliaceus* sp. nov.. Populations of the new species (red stars) and the city center of Municipality of Santa Teresa (yellow circle), southeastern Brazil. BA (State of Bahia), ES (State of Espírito Santo), MG (State of Minas Gerais), and RJ (State of Rio de Janeiro).

**Distribution.** *Dendropsophus bromeliaceus* sp. nov. is currently only known from three rocky outcrops in the area surrounding the Reserva Biológica Augusto Ruschi in the Municipality of Santa Teresa, a mountainous region of the State of Espírito Santo, southeastern Brazil (Fig 6). The new species was not found in any of the seven forested sites investigated.

**Natural history.** *Dendropsophus bromeliaceus* sp. nov. has exclusively been found on rocky outcrops with sparse trees of low to medium heights; the ground covered with dense layer of bromeliads and herbaceous plants. Epiphytic bromeliads almost completely covering the tree branches. This vegetation pattern is distinct from that of the surrounding forested areas, which are shaded due to higher densities of large trees.

*Dendropsophus bromeliaceus* sp. nov. is a nocturnal frog with males calling in both the rainy (October through December) and dry season (June and July). However, choruses were less pronounced during the dry season with fewer individuals calling and less frequent calls. Tadpoles and juvenile froglets were only found in the rainy season. No amplectant pair or eggs were found during our surveys.

We recorded natural history observations of 12 adults, four juvenile froglets, and 10 exotrophic tadpoles of *Dendropsophus bromeliaceus* sp. nov. found in the rainwater accumulated inside bromeliads (Table 2). Calling males and tadpoles were in bromeliads located on the ground and up to 5 m above ground. Males called from horizontal leaves outside the axils of bromeliads. All adults, froglets, and tadpoles were found in the median axils (i.e. basal tank and central axils were not used). Four calling males were collected from bromeliads containing no tadpole or froglet. Another three calling males were in bromeliads with conspecific tadpoles or froglets. The other five adult individuals were found in bromeliads that did not harbor tadpoles or juvenile froglets.

**Table 2:** Characteristics of bromeliad species used by *Dendropsophus bromeliaceus* sp. nov.. N = number of bromeliads; PD = plant diameter in cm; PH = plant height in cm; NL = number of leaves; PS = plant height from the soil in m. Frogs (sex or life stage) per bromeliad: A = adult (n = 3), M = calling male (n = 8), F = froglet (n = 4), T = tadpole (n = 10); + indicates frogs were in the same bromeliad; / indicates frogs were in different bromeliads. Mean and standard deviation are provided when appropriate.

Bromeliad species	N	PD	PH	NL	PS	Frogs
*Aechmea capixabae*	1	31	28	14	1.5	M+2T+F
*Aechmea lamarchei*	1	60	67	13	0	T
*Aechmea pineliana*	1	70	64	23	0	T
*Alcantarea extensa*	1	56	62	22	0	M
*Neoregelia pauciflora*	1	18	15	12	2.0	M+T
*Racinaeae spiculosa*	3	22 ± 7.8	21 ± 2.6	17 ± 7	2.3 ± 1.0	A/M/M
*Vriesea bituminosa*	3	14.3 ± 4.7	13.3 ± 4.2	10.7 ± 3.1	2.5 ± 2.2	F+2T/A/T
*Vriesea morrenii*	2	18.7 ± 2.5	20 ± 2.8	22 ± 2.8	1.85 ± 0.2	M/2T
*Vriesea ruschii*	4	58.25 ± 19.8	37 ± 12.1	16.8 ± 2.1	0	M+2F/M/A/F
Total	17	38.7 ± 20.1	36.4 ± 20.9	16.7 ± 4.4	1.1 ± 1	

*Vriesea ruschii* is the dominant bromeliad on the outcrops, and was also the most commonly used plant by *D*. *bromeliaceus* sp. nov. (Table 2). The terrestrial bromeliads used by *D*. *bromeliaceus* sp. nov.had a wider diameter (F_1,15_ = 58.92; *P* < 0.001) and greater height (F_1,15_ = 28.12; *P* < 0.001) than the epiphytic bromeliads at the sites. A number of bromeliad species were present at the sites but not occupied by *D*. *bromeliaceus* sp. nov., including *Bilbergia* sp., *Edmundoa lindenii*, *Quesnelia strobilispica*, *Neoregelia macrosepala*, *Neoregelia* sp., *Nidularium cariacicaense*, *Nidularium espiritosantense*, *Nidularium* sp., *Vriesea* aff. *atra*, *V*. *ensiformis*, and *V*. *vagans*.

Adults of *Dendropsophus bromeliaceus* sp. nov. were not found in the same bromeliad with congeners. On one occasion, *D*. *bromeliaceus* sp. nov. shared the same plant (*Alcantarea extensa*) with another frog species (*Thoropa miliaris*), but they used different axil positions; *D*. *bromeliaceus* sp. nov. was in a median axil whereas *T*. *miliaris* was in a basal axil. Although *D*. *bromeliaceus* sp. nov. and *Scinax arduous* were the most abundant frogs at these sites and were frequently found in *Vriesea ruschii*, they did not share the same individual plant. In total, we found the following 12 frog species in syntopy with *D*. *bromeliaceus* sp. nov. inside bromeliads: *Bokermannohyla caramaschii*, *F*. *fissilis*, *Fritziana goeldii*, *Gastrotheca megacephala*, *Hypsiboas pardalis*, *H*. *semilineatus*, *Ischnocnema abdita*, *I*. *epipeda*, *I*. cf. *parva*, *Scinax alter*, *S*. *arduous* and *Thoropa miliaris*.

## Discussion

The monophyly of *Dendropsophus* is supported by diferent lines of evidence (e.g. morphology and cytogenetic) [22,82], but the backbone of the *Dendropsophus* phylogenetic tree remains largely unresolved. Further phylogenetic studies of the genus are needed to clarify the relationships among the major clades.

The strict dependence of *Dendropsophus bromeliaceus* sp. nov. upon bromeliads to complete its life cycle (reproductive mode = 6, sensu [83]) is an exceptional trait in *Dendropsophus*. In addition to still water, some species of *Dendropsophus* can use alternative habitats for depositing their eggs. For example, *D*. *ebraccatus* can lay eggs on vegetation over water bodies, or lay eggs directly in the water [84]; the pond breeder *D*. *haddadi* may sometimes lay eggs in bromeliads, but no tadpoles or froglets of this species have ever been observed inside bromeliad axils, likely making this reproductive mode only facultative in this species [85]. Considering the known–or inferred–oviposition mode in genera closely related to *Dendropsophus* as well as the distribution of this mode in *Dendropsophus*, it is more parsimonious to assume that oviposition in still water as a plesiomorphic trait in *Dendropsophus*. *Dendropsophus bromeliaceus* sp. nov. used several bromeliad species with a wide range of characteristics (Table 2). However, the new species seems to avoid many bromeliad species, possibly because they have only a single central tank, or are not able to store enough rainwater. *Dendropsophus bromeliaceus* sp. nov. possibly avoids central tanks and basal axils, due to the risk of desiccation, predation, and/or disturbance.

The low number of tadpoles per plant (one or two) may indicate that *D*. *bromeliaceus* sp. nov. deposits only few eggs per bromeliad, or that tadpoles are cannibalistic. Some bromeligenous frogs are known to lay a reduced number of eggs as a way to avoid competition among tadpoles [86]. In other species, tadpoles subsist on the eggs of their own or other species of frogs [87,88].

*Dendropsophus bromeliaceus* sp. nov. may exhibit tadpole guarding if the tadpoles found in bromeliads with adults prove to be their own progeny; this needs further investigation. Several bromeligenous species exhibit parental care, which is possibly a response to evolutionary pressure imposed by the harsh environment and resource limitation inside bromeliads [86].

Our field observations suggest that *D*. *bromeliaceus* sp. nov. is an intraspecific territorial species. This behavior is not unusual in bromeligenous frogs, which select and defend their oviposition microhabitats against conspecifics (e.g. *Phyllodytes luteolus* [89,90]; *Crossodactylodes izecksohni*, RBF pers. obs.).

**Conservation remarks**: The most distant populations of *Dendropsophus bromeliaceus* sp. nov. were about 13.5 km straight line apart. All the rocky outcrops studied are on private property surrounding the REBIO Augusto Ruschi. Based on the known distribution of *Dendropsophus bromeliaceus* sp. nov., its extent of occurrence is about 50 km^2^. Although *D*. *bromeliaceus* sp. nov. could be listed as Critically Endangered (CR) under IUCN criteria B1a,b and B2a,b [91], at this time it should be listed as Data Deficient due to the lack of information on its exact geographic distribution and population size. It is likely that this species occurs more widely, and possibly inside the REBIO Augusto Ruschi (ca. 3591 ha).

The fact that the current three populations are only known from private property highlights the vital importance of preserving these natural areas. In addition, they function as forest corridors for several species [92]. An outreach environmental education program should be implemented by the federal, state, and local agencies to protect these populations. Furthermore, because rocky outcrops do not attract much agricultural interest, they have frequently been preserved from human impact, which up to now may have helped the new species to survive [10].

Despite the fact that rocky outcrops may not be converted to agricultural land, bromeliads are commonly collected by local people for yard decorations (RBF, pers. obs.). *Dendropsophus bromeliaceus* sp. nov. used two bromeliad species (*Aechmea capixabae* and *Vriesea morrenii*) currently listed as vulnerable to extinction, in part due to overexploitation, but also due to habitat loss [93]. Illegal collection of these bromeliad species for ornamental purposes may affect bromeligenous frogs across rocky outcrops. Regulation regarding collection of these plants should be implemented to avoid overexploitation.

The discovery of this new species emphasizes the importance of this mountainous region for amphibian conservation. Even though Santa Teresa and its surrounding areas in southeastern Brazil are one of the most sampled regions in the Atlantic Forest, the region still harbors numerous remote areas that have not yet been sampled for frogs. The region is an important hotspot for anuran and bromeliad conservation due to its high richness and number of endemic species [94,95,96,97]. By including this new species and *Chiasmocleis schubarti* (João F. R. Tonini pers. comm.) to the list of its herpetofauna, Santa Teresa harbors 94 recognized frog species [94,95]. The area also harbors 107 bromeliad species [96]. The rate of new species discoveries suggests Santa Teresa’s biodiversity is far from fully described.

## Supporting Information

S1 File Additional specimens examined. (DOCX)
