# Supplementary material for: The First Bromeligenous Species of Dendropsophus (Anura: Hylidae) from Brazil's Atlantic Forest
Source: PLoS One. 2015 Dec 9;10(12):e0142893. doi: 10.1371/journal.pone.0142893 (PMC4674083; doi:10.1371/journal.pone.0142893)
Supplement: S1 File — (DOCX) [file pone.0142893.s001.docx]

**S1 File. Additional specimens examined.** (DOC)

*Dendropsophus acreanus:* Brazil: Acre: Tarauacá (MNRJ 3971).

*Dendropsophus anceps:* Brazil: Rio de Janeiro: Cachoeira de Macacu (MNRJ 86722-25).

*Dendropsophus araguaya:* Brazil: Mato Grosso: Alto Araguaia (MNRJ 17240-41) (paratypes).

*Dendropsophus* *berthalutzae*: Brazil: Rio de Janeiro: Duque de Caxias (MNRJ 85196-200); Rio de Janeiro (MNRJ 75053-55).

*Dendropsophus* *bipunctatus*: Brazil: Bahia: Ubaitaba (MNRJ 51623-31); Rio de Janeiro: Búzios (MNRJ 82904-23).

*Dendropsophus* *branneri*: Brazil, Bahia: Ilhéus (MNRJ 34356-88).

*Dendropsophus* *cachimbo*: Brazil: Pará: Cachimbo (MNRJ 17298-99) (paratypes).

*Dendropsophus* *cerradensis*: Brazil: Mato Grosso do Sul: Ribas do Rio Pardo (MNRJ 17293) (paratype).

*Dendropsophus cruzi*: Brazil: Goiás: Mossâmedes (MNRJ 21801-802, 21799-800); Silvânia (MNRJ 21782) (holotype), (MNRJ 18215-16) (paratypes).

*Dendropsophus* *decipiens*: Brazil: Rio de Janeiro: Itaguaí (MNRJ 62525-78).

*Dendropsophus elegans*: Brazil: Espírito Santo: Santa Teresa (MNRJ 30443-44); Rio de Janeiro: Jurubatiba: (MNRJ 66439-40).

*Dendropsophus elianeae*: Brazil: Mato Grosso: Cáceres (MNRJ 17194-99); Mato Grosso do Sul: Bela Vista (MNRJ 17297) (holotype), (MNRJ 17226-34) (paratypes).

*Dendropsophus* *giesleri*: Brazil: Rio de Janeiro: Magé (MNRJ 55244); Nova Iguaçu (MNRJ 86466-89).

*Dendropsophus haddadi*: Brazil: Espírito Santo: Conceição da Barra (MNRJ 17325) (holotype); Linhares (MNRJ 17078-82) (paratypes).

*Dendropsophus jimi*: Brazil: São Paulo: Botucatu (MNRJ 21980) (holotype), (MNRJ 21981-89) (paratypes).

*Dendropsophus labialis*: Colombia: Paramo Bogota (MNRJ 49772).

*Dendropsophus* *leali*: Brazil: Rondônia: Forte Príncipe da Beira (MNRJ 3962) (paratype).

*Dendropsophus* *leucophyllatus*: Brazil: Tocantins: Araguaína (MNRJ 88535-37).

*Dendropsophus* *marmoratus*: Brazil: Amazonas: Barcelos (MNRJ 36241-42).

*Dendropsophus* *melanargyreus*: Brazil: Pará: Tucuruí (MNRJ 17786).

*Dendropsophus* *meridianus*: Brazil: Rio de Janeiro: Itaguaí (MNRJ 62406-76).

*Dendropsophus* *microcephalus*: Colombia: Girardot (MNRJ 21834-40); Costa Rica: Puntarenas (MNRJ 3641, 14636-39).

*Dendropsophus* *microps*: Brazil: São Paulo: São José do Barreiro (MNRJ 76654-57).

*Dendropsophus* *minutus*. Brazil: Minas Gerais: Sacramento (MNRJ 88598-609); Rio de Janeiro: Nova Friburgo (MNRJ 77141-43); São Paulo: Botucatu (MNRJ 65240-88).

*Dendropsophus* *nahdereri*: Brazil, Santa Catarina: São Bento do Sul (MNRJ 3295) (lectotype), (MNRJ 3294, 3296) (paralectotype).

*Dendropsophus* *nanus*: Brazil: São Paulo: Botucatu (MNRJ 80017, 81397).

*Dendropsophus* *novaisi*: Brazil: Bahia: Maracás (MNRJ 4049) (paratype).

*Dendropsophus* *oliveirai*: Brazil: Bahia: Maracás MNRJ 3668).

*Dendropsophus ozzyi*: Brazil: Pará: Juruti (MNRJ 86921-25) (paratypes).

*Dendropsophus phlebodes*: Costa Rica: Alajuela (MNRJ 3639, 14635).

*Dendropsophus pseudomeridianus*: Brazil: Rio de Janeiro: Seropédica (MNRJ 25502) (holotype), (MNRJ 25503-32) (paratypes).

*Dendropsophus* *rhea*: Brazil: São Paulo: Pirassununga (MNRJ 17241-46) (paratypes)

*Dendropsophus* *rhodopeplus*: Ecuador: Prov. Pastaza. (MNRJ 73360-61).

*Dendropsophus rossalleni*: Brazil: Amazonas: Itacoatiara, Cairiri (MNRJ 56787-91).

*Dendropsophus* *rubicundulus*: Brazil: Minas Gerais: Catas Altas (MNRJ 60611-14).

*Dendropsophus* *ruschii*: Brazil: Espírito Santo: Pedra Azul (MNRJ 31548-50); Minas Gerais: Pedra Dourada: (MNRJ 47849-55, 478457).

*Dendropsophus sanborni*: Brazil: Estado de São Paulo: Ribeirão Branco (MNRJ 18210-11).

*Dendropsophus* *schubarti*: Brazil: Rondônia: (MNRJ 3669) (holotype).

*Dendropsophus* *seniculus*: Brazil: Espírito Santo: Cariacica (MNRJ 27910-12); Rio de Janeiro: Jurabatiba (MNRJ 88048-58).

*Dendropsophus* *soaresi*: Brazil: Piauí: Picos (MNRJ 60083) (holotype).

*Dendropsophus* *tritaeniatus*: Brazil: São Paulo: Pirajú (MNRJ 17225).

*Dendropsophus* *walfordi*: Brazil: Amazonas: Lago Janauacá (MNRJ 18141-44).

*Dendropsophus werneri*: Brazil, Paraná, Guaraqueçaba (MNRJ 15608-10), (MNRJ 21843-44); Santa Catarina: Joinvile (MNRJ 1542, 8201-03, 8205, 8207-13); Santa Luzia (MNRJ 2099, 10639-40).
